# Immunogenic Cell Death Traits Emitted from Chronic Lymphocytic Leukemia Cells Following Treatment with a Novel Anti-Cancer Agent, SpiD3

**DOI:** 10.3390/biomedicines12122857

**Published:** 2024-12-16

**Authors:** Elizabeth Schmitz, Abigail Ridout, Audrey L. Smith, Alexandria P. Eiken, Sydney A. Skupa, Erin M. Drengler, Sarbjit Singh, Sandeep Rana, Amarnath Natarajan, Dalia El-Gamal

**Affiliations:** 1 Eppley Institute for Research in Cancer and Allied Diseases, University of Nebraska Medical Center, Omaha, NE 68198, USA; eschmitz@unmc.edu (E.S.); audrey.smith@unmc.edu (A.L.S.); alexandria.eiken@vai.org (A.P.E.); sydney.skupa@unmc.edu (S.A.S.); edrengler@unmc.edu (E.M.D.); sarbjit.dhami@gmail.com (S.S.); sandeep.rana@nih.gov (S.R.); anatarajan@unmc.edu (A.N.); 2 Fred and Pamela Buffett Cancer Center, University of Nebraska Medical Center, Omaha, NE 68198, USA

**Keywords:** chronic lymphocytic leukemia (CLL), SpiD3, immunogenic cell death (ICD), damage-associated molecular patterns (DAMPs), ferroptosis, oxidative stress, cancer immunity

## Abstract

**Background:** Targeted therapies (e.g., ibrutinib) have markedly improved chronic lymphocytic leukemia (CLL) management; however, ~20% of patients experience disease relapse, suggesting the inadequate depth and durability of these front-line strategies. Moreover, immunotherapeutic success in CLL has been stifled by its pro-tumor microenvironment milieu and low mutational burden, cultivating poor antigenicity and limited ability to generate anti-tumor immunity through adaptive immune cell engagement. Previously, we have demonstrated how a three-carbon-linker spirocyclic dimer (SpiD3) promotes futile activation of the unfolded protein response (UPR) in CLL cells through immense misfolded-protein mimicry, culminating in insurmountable ER stress and programmed CLL cell death. **Method:** Herein, we used flow cytometry and cell-based assays to capture the kinetics and magnitude of SpiD3-induced damage-associated molecular patterns (DAMPs) in CLL cell lines and primary samples. **Result:** SpiD3 treatment, *in vitro* and *in vivo*, demonstrated the capacity to propagate immunogenic cell death through emissions of classically immunogenic DAMPs (CALR, ATP, HMGB1) and establish a chemotactic gradient for bone marrow-derived dendritic cells. **Conclusions:** Thus, this study supports future investigation into the relationship between novel therapeutics, manners of cancer cell death, and their contributions to adaptive immune cell engagement as a means for improving anti-cancer therapy in CLL.

## 1. Introduction

Typically presenting through the indolent accumulation of CD5+ B-cells in the blood, bone marrow, and secondary lymphoid structures, chronic lymphocytic leukemia (CLL) is categorized as a type of B-cell non-Hodgkin lymphoma resulting in clinically notable lymphocytosis, lymphadenopathy, and splenomegaly [1,2,3]. Immunotherapeutic success in CLL has been stifled by a pro-tumor microenvironment and a low mutational burden, cultivating poor antigenicity and the limited ability to generate anti-tumor immunity through adaptive immune cell engagement. Thus, therapeutics, targeted or otherwise, which improve the antigenicity or adjuvanticity of CLL cells may be necessary to eradicate the CLL disease burden.

Anti-cancer therapy has long been focused on eradicating cancer cells by directing therapeutic agents towards cancer-specific targets. Revelations in CLL biology and advancements in molecular profiling have led to better patient stratification and to the discovery of CLL-specific targets (e.g., BTK, PI3K, BCL-2), prompting CLL’s own targeted small-molecule inhibitor era, led by the Bruton’s tyrosine kinase (BTK) inhibitor, ibrutinib, which has demonstrated durable responses in both front-line and relapsed/refractory settings in addition to the treatment of high-risk disease patients (e.g., TP53 alterations) [3,4,5,6]. However, many CLL patients eventually discontinue ibrutinib due to disease progression or toxicity, necessitating alternative therapeutic strategies.

Researchers have demonstrated B-cell receptor (BCR) signaling as a central driver for CLL, in addition to other contributors such as aberrant anti-apoptotic (e.g., BCL-2, TRAIL, MCL-1), pro-survival (e.g., IL-4R and IL-21R), and cell-trafficking (e.g., CCR7 and CXCR4) proteins. Novel anti-cancer agents aimed at these aberrations have been developed with promising pre-clinical and clinical data [7,8,9,10,11,12,13,14,15]. Nonetheless, the novelty of a target does not equate to the depth and durability of the response. Cancer cell death remains a primary goal for most anti-cancer therapies; however, cancer cell death is also the first step in the cancer-immunity cycle, a process that defines the progression of antigen acquisition to immunologic memory. Hence, improving the depth and durability of CLL-directed therapeutics may require the consideration of not only the intended target to provoke CLL cell death, but the immunologic consequences of the death elicited.

Cell injury, damage, or death is communicated to immune cells through damage-associated molecular patterns (DAMPs) and their subsequent interactions with the pattern recognition receptors (PRRs) of immune cells. DAMPs convey a tolerogenic (immunosuppressive) or immunogenic (immunostimulatory) response; it is the balance of both tolerogenic, and immunogenic DAMPs emitted by a cell death mechanism that ultimately shapes the immuno-potential of the cellular microenvironment [16,17]. Independent of the cell death mechanism (e.g., apoptosis, pyroptosis, necrosis, ferroptosis), immunogenic cell death (ICD) describes a context in which the DAMPs emitted from a dying cell (e.g., CALR, ATP, HMGB1) cultivate a net immunostimulatory microenvironment, perpetuating the cancer-immunity cycle and curating an antigen-specific immune response. A shared characteristic of *bona fide* ICD inducers is their ability to provoke endoplasmic reticulum (ER) stress and generate reactive oxygen species (ROS) [18].

In collaboration with colleagues, we previously characterized α-methylene-γ-butyrolactone-containing spirocyclic dimers (SpiDs) as novel, anti-cancer agents for B-cell malignancies such as CLL [19,20,21,22,23]. SpiDs covalently modify NF-κB proteins, P65, and IKKβ, by the targeting of surface-exposed cysteine residues, ultimately inducing a futile unfolded protein response (UPR) [20,21]. The UPR is an evolutionarily advantageous mechanism used to contend, and ultimately restore, disruptions to proteostasis through the upregulation of ER chaperones (thus increasing protein folding capacity) and ER-associated degradation proteins (thus clearing misfolded proteins), thereby resolving ER stress and restoring proteostasis. However, when the UPR proves futile under conditions of overwhelming or prolonged ER stress, cells activate programmed cell death pathways, leading to their demise. We have investigated how a three-carbon-linker SpiD (SpiD3) promotes futile activation of the UPR in CLL cells through immense misfolded-protein mimicry, culminating in insurmountable ER stress and programmed cell death [20]. Our previous work demonstrated that SpiD3-related apoptotic cell death was partially recovered when CLL cells were pre-treated with the pan-caspase inhibitor, z-VAD-FMK, suggesting that additional mechanisms of cell death may contribute to SpiD3’s cytotoxicity [20]. A further investigation demonstrated that SpiD3-mediated CLL cell death may partly rely on ferroptosis and peroxidized lipid accumulation [19]. In this study, we hypothesize that this insurmountable ER stress and ROS generation may confer SpiD3 the unique ability to promote immunogenic DAMP release for ICD propagation in CLL cells. We used CLL cell lines and primary samples to demonstrate the magnitude and kinetics of DAMP emissions in an “immunologically cold” cancer through therapeutic induction of immunologically relevant adjuvants to potentiate the cancer-immunity cycle.

## 2. Materials and Methods

### 2.1. Pharmacological Agents

SpiD3 was synthesized by the Natarajan lab at the University of Nebraska Medical Center (UNMC; Omaha, NE, USA) following reported procedures [21,22,23]. Etoposide (Cat #12092) and ferrostatin-1 (Cat #17729) were purchased from Cayman Chemical (Ann Arbor, MI). Iron (II) chloride tetrahydrate (FeCl_2_·4H_2_O) was purchased from Thermo Scientific Chemicals (Cat #205080050; Waltham, MA, USA). SpiD3 and etoposide were dissolved in DMSO. Compounds and vehicle controls were administered so that the final DMSO concentration was <0.1% in cell cultures. Ferrostatin-1 and FeCl_2_ were reconstituted in a solution of sterile water containing 0.1% bovine serum albumin.

### 2.2. CLL Cell Lines

The HG-3 cell line was ordered from DSMZ (Braunschweig, Germany). The OSU-CLL cell line was provided by the Human Genetics Sample Bank of The Ohio State University (Columbus, OH, USA) [24]. All CLL cell lines were cultured at 37 °C in complete RPMI-1640 media with the addition of 2 mM L-glutamine (Cat #R8758; Sigma-Aldrich; St. Louis, MO, USA), 100 U/mL penicillin, 100 μg/mL streptomycin (P/S; Cat #P0781; Sigma-Aldrich), and 10% heat-inactivated fetal bovine serum (hi-FBS; Cat #97068-091; Avantor^®^; Radnor, PA, USA). All cell cultures were passaged every 48–72 h to maintain optimal cell densities. Before experimental use, cell line cultures were confirmed to be free of mycoplasma contamination using the luminescence-based MycoAlert^®^ Mycoplasma Detection Kit (Lonza Cat #LT07-318; Basel, Switzerland).

### 2.3. Primary CLL Cells

Peripheral blood mononuclear cells (PBMCs) were isolated from CLL patient blood using Lymphoprep™ (Cat #07851; StemCell Technologies; Vancouver, BC, Canada) density gradient centrifugation following the manufacturer’s protocols. The CLL patient-derived PBMCs used in this study were verified to contain >90% CD45+/CD19+/CD5+ cells. CLL patient sample characteristics are tabulated in Supplementary Table S1. Cells were maintained at 37 °C in RPMI-1640 with 2 mM L-glutamine, supplemented with 10% hi-FBS and P/S.

### 2.4. Surface CALR Expression

HG-3 and OSU-CLL cell lines (1 × 10^6^ cells/mL) or CLL patient-derived PBMCs (2 × 10^6^ cells/mL) were treated with the vehicle (DMSO), SpiD3 (0.5–2 µM), etoposide (20 µM), or 160 µM iron (II) chloride tetrahydrate (FeCl_2_) for up to 48 h at 37 °C. Treatment-induced changes in surface CALR expression were evaluated via flow cytometry using Calreticulin (D3E6) XP^®^ Rabbit mAb (PE Conjugate; Cat #19780; Cell Signaling Technologies; Danvers, MA, USA) and Zombie NIR Fixable Viability Dye (Cat #423106; BioLegend; San Diego, CA, USA). Primary patient-derived CLL cells and Eμ-TCL1 splenic CLL cells were classified as CD19+/CD5+ using the following antibodies: FITC anti-human CD19 Antibody (Cat #302206; BioLegend); APC anti-human CD5 Antibody (Cat #364016; BioLegend); Brilliant Violet 711™ anti-mouse CD5 Antibody (Cat #100639; BioLegend); and BD Horizon™ BUV395 Rat Anti-Mouse CD19 (Cat #563557; BD Biosciences; Franklin Lakes, NJ, USA). The percentage of CALR+ viable cells compared to that of the vehicle-treated condition was identified in comparison to fluorescence minus one (FMO) controls, wherein the cells were only stained with viability dye and antibodies other than CALR-PE.

### 2.5. Extracellular ATP Measurement

Extracellular ATP was kinetically monitored in CLL cell lines over a 24 h period using the RealTime-Glo™ Extracellular ATP Assay (Cat #GA5010; Promega; Madison, WI, USA) following the manufacturer’s instructions. Due to the nature of the plate reader used (POLARstar OPTIMA; BMG LABTECH), cells and reagents were prepared in CO_2_-independent media (Leibovitz’s L-15 Medium; Cat #11415064; Gibco; Waltham, MA, USA). HG-3 and OSU-CLL cell lines were seeded at 2.5 × 10^5^ cells in 100 µL per well in a luminescence-grade 96-well plate (Cat #655095; Greiner-Bio; Monroe, NC, USA), then subjected to 50 µL of 4X treatment with the vehicle (DMSO), SpiD3 (0.5–2 µM), or etoposide (20 µM), immediately followed by 50 µL of 4X RealTime-Glo™ Extracellular ATP Assay Reagent. The average luminescence of the replicates was plotted as a function of treatment condition over time. Extracellular ATP levels at specific time points were further evaluated in comparison to the timepoint-matched vehicle.

### 2.6. Extracellular HMGB1 Detection

HG-3 and OSU-CLL cell lines (1 × 10^6^ cells/mL in 24-well plate) or CLL patient-derived PBMCs (0.5 × 10^6^ cells/mL in 96-well plate) were treated with vehicle (DMSO), SpiD3 (0.5–2 µM), or etoposide (20 µM) for up to 48 h at 37 °C. Following treatment, cells were centrifuged at 350× *g* for 7 min to isolate the supernatant. Each treated CLL cell supernatant (80 μL) was then transferred to a luminescence-grade 96-well plate (Cat #655095; Greiner-Bio) and extracellular HMGB1 was detected using the Lumit™ HMGB1 Immunoassay (Cat #W6110; Promega) per the manufacturer’s instructions. Luminescence was monitored on a Tecan Infinite^®^ M1000 Pro microplate reader (Männedorf, Switzerland).

### 2.7. Bone-Marrow Derived Dendritic Cell (BMDC) Migration

A CD11c+ murine dendritic cell population was generated *ex vivo* by harvesting murine bone marrow from the long bones (femurs and tibiae) of wild-type C57BL/6J (WT B6) mice (Strain #000664; The Jackson Laboratory; Bar Harbor, ME, USA) and culturing in RPMI-1640 media, supplemented with a cytokine cocktail of 20 ng/mL mouse recombinant granulocyte–macrophage colony-stimulating factor (GM-CSF; Cat #78017; StemCell Technologies) and 20 ng/mL mouse recombinant IL-4 (Cat #78047.1; StemCell Technologies) for 6 days following established protocols [25,26,27]. All WT B6 mice used for the experiments were between 4 and 7 months of age and were maintained at the UNMC animal facility.

The chemotactic effects of SpiD3-induced DAMPs on bone marrow dendritic cells (BMDCs) were evaluated using a Transwell migration assay. On day 5 of the BMDC culture, HG-3 and OSU-CLL cells were seeded at 1 × 10^6^ cells/mL in a 24-well plate, then subjected to treatment with the vehicle (DMSO), SpiD3 (0.5–2 µM), or etoposide (20 µM) for the following 24 h at 37 °C. After the incubation period (on day 6 of the BMDC culture), 400 µL of the supernatant from each treatment condition was plated in the bottom chamber of a 24-well Transwell migration plate (Cat #3415; Corning; Corning, NY, USA). BMDCs were loaded into the Transwell insert and allowed to migrate toward the supernatant below over 6 h of undisturbed incubation at 37 °C. RPMI-1640 supplemented with 20 ng/mL mouse recombinant GM-CSF (Cat #78017; StemCell Technologies) and supernatant derived from heat-shocked CLL cells were used as positive migration controls. After 6 h, the Transwell insert was carefully removed and the number of cells that migrated to the bottom chamber was evaluated via flow cytometry. The chemotactic index of each condition was calculated by dividing the migrated cell count observed by that observed in the vehicle-treatment condition.

### 2.8. Eμ-TCL1 Spleen and Plasma Analysis

In a previous study performed by Eiken, et al. [20], Eµ-TCL1 mice with comparable leukemia (median leukemia burden of 51.8% CD19+/CD5+ peripheral blood lymphocytes) were treated with the SpiD3 prodrug (SpiD3_AP, 10 mg/kg) or vehicle equivalent (50% PEG400, 10% DMSO, 40% water) intravenously once daily for 3 days. Approximately 3 h after administration of the final dose, mice were anesthetized with isoflurane (VetOne Cat #502017; Paris, France) and blood was obtained via cardiac puncture. Following euthanasia, murine spleens were harvested and homogenized into a single-cell suspension by passing through a 70 μm filter. Red blood cells were lysed (RBC Lysis Buffer; Cat #420301; BioLegend) prior to staining for flow cytometry. Blood was centrifuged at 2000× *g* for 15 min at 4 °C to isolate the plasma layer, and plasma was stored at −80 °C until use. The concentrations of plasma inflammatory cytokines and chemokines were assessed using BioLegend LEGENDplex™ flow cytometry-based multiplex immunoassays per the manufacturer’s protocols. The following analytes were evaluated on undiluted plasma using the Mouse Anti-Virus Response Panel (Cat #740622; BioLegend): IFN-γ; IFN-α; IL-1β; IL-12p70; GM-CSF; CXCL10; and TNF-α. The following analytes were evaluated on 2-fold diluted plasma using the Mouse Pro-Inflammatory Chemokine Panel (Cat #740451; BioLegend): CCL2; CCL3; CCL4; CCL5; CCL11; CCL17; CXCL9; and CXCL13.

### 2.9. Flow Cytometry

Cells (~1–2 × 10^6^) were incubated in 100 μL PBS 2% hi-FBS containing fluorochrome-labelled antibodies for 30 min at 4 °C. When required, labeled cells were fixed using BioLegend Fixation Buffer (Cat #420801) per the manufacturer’s protocol. Flow cytometry was performed on a NovoCyte 2060R (Agilent; Santa Clara, CA, USA) or BD LSRII (BD Biosciences) cytometer. All flow cytometry data were analyzed using NovoExpress v1.3.0 (Agilent) or Kaluza v2.1 (Beckman Coulter; Brea, CA, USA). Specific staining panels and gating strategies are described, where appropriate, within the methodology above and in the Supplementary Methods.

### 2.10. Statistics

Data are reported as mean ± standard error of the mean (SEM). The statistical significance between two groups was determined via unpaired *t*-tests coupled to post hoc analysis with Welch’s correction. The statistical significance of more than two groups was analyzed by one-way ANOVA with Dunnett’s multiple comparisons post hoc analysis. All statistical analyses were conducted by GraphPad Prism v9.4.1 (GraphPad Software; Boston, MA, USA). *p* values less than 0.05 were considered significant.

## 3. Results

### 3.1. SpiD3 Treatment Mediates Immunogenic DAMP Release from CLL Cells

Due to distinct DAMP emissions, ICD is predicated by a temporal sequence of changes in cell surface composition concurrent to the specific release of soluble mediators [16,17,28,29]. Observable changes include the translocation of proteins to the dying cell’s surface and the secretion of soluble mediators, which serve as ligands for the PRRs expressed by monocytes, macrophages, dendritic cells (DCs), and other antigen-processing cells.

#### 3.1.1. SpiD3-Treated CLL Cells Display Ecto-CALR

**Figure 1:**
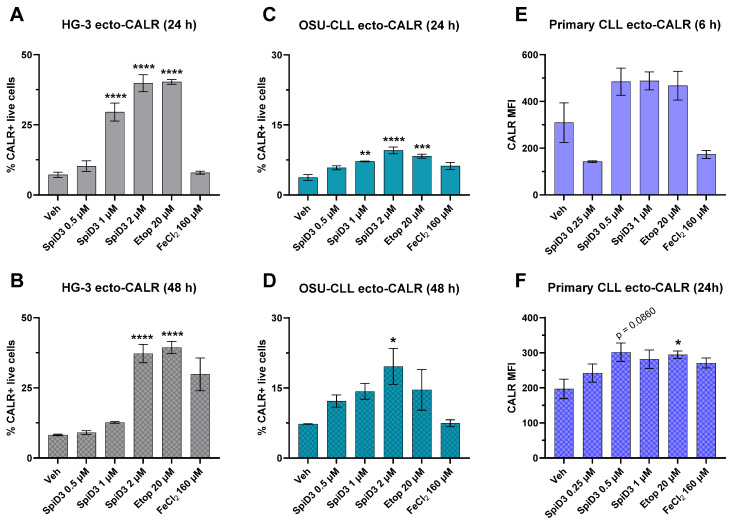
CLL cells display ecto-CALR following SpiD3 treatment. HG-3 ((**A**,**B**); n = 3); OSU-CLL ((**C**,**D**); n = 3); or patient-derived CLL ((**E**,**F**); n = 5) cells were treated with vehicle (Veh), SpiD3 (0.25–2 µM), FeCl_2_ (160 μM), or the positive control, etoposide (Etop; 20 µM) for the indicated durations. Viable cells were analyzed by flow cytometry for changes in surface CALR expression (ecto-CALR). Primary patient-derived CLL cells were additionally designated as CD19+/CD5+ by flow cytometry. Data are presented as mean ± SEM. Comparisons across treatment groups were analyzed with respect to the vehicle by one-way ANOVA. Asterisks denote magnitude of significance: * *p* < 0.05, ** *p* < 0.01, *** *p* < 0.001, **** *p* < 0.0001.

Calreticulin (CALR), one of the better-studied DAMPs, serves as an “eat me” signal for DCs [30,31]. CALR is a resident ER chaperone protein, and, as such, is normally secluded in the ER lumen. Under certain cellular stressors, CALR is translocated to the surface of the cell’s plasma membrane (ecto-CALR), where it can signal phagocytes [30,31,32,33,34,35,36]. Using a fluorescent-labelled anti-CALR antibody in combination with a viability dye, the translocation of CALR to the surface membranes of viable cells was detected by flow cytometry. The HG-3 CLL cell line demonstrated intense ecto-CALR expression following 24 h of treatment, starting at the 1 µM SpiD3 concentration and emboldened by the 2 µM SpiD3 concentration, in a manner comparable to that of the positive control, etoposide (Figure 1A). Despite the strong lipid peroxidation effect of 160 µM FeCl_2_ treatment (Supplementary Figure S1A), intense peroxidized lipid accumulation does not appear to induce CALR surface translocation in HG-3 cells (Figure 1A). When SpiD3 treatment of HG-3 cells was extended to 48 h, we observed curtailed CALR translocation from the 1 µM SpiD3 treatment group, consistent with literature suggesting that ecto-CALR is an early ICD event [31,37]. This may be further explained by the limited viable cells that remained at the 48 h timepoint to contribute to the CALR+ population [20]. Consequently, in potent treatment conditions (i.e., 2 µM SpiD3 and etoposide), cellular stress was sustained among the remaining cell population, producing a CALR+ population at the 48 h timepoint, despite diminished cell viability (Figure 1B). Interestingly, prolonged exposure to 160 µM FeCl_2_ treatment culminated in sufficient cellular stress for CALR translocation, comparable to the potent conditions of 2 µM SpiD3 and etoposide, implying that acute CALR translocation (ecto-CALR prior to 48 h) requires additional cellular stressors for HG-3 cells than the generation of peroxidized lipids alone (Figure 1B). OSU-CLL cells display enhanced CALR surface expression at both 24 h and 48 h timepoints in an SpiD3 dose-dependent manner (Figure 1C,D), with CALR translocation induced most prominently by cells treated with 2 µM SpiD3 for 24 h (Figure 1C). In contrast to HG-3 cells, OSU-CLL cells demonstrate statistically significant ecto-CALR within 24 h of 160 µM FeCl_2_ treatment, suggesting the kinetics of CALR surface translocation is partly cell line dependent. SpiD3 treatment also yielded increased ecto-CALR expression on primary patient-derived CLL cells, similar to that evoked by etoposide at 6 h and 24 h (Figure 1E,F). Together, these data show that SpiD3 treatment enhanced ecto-CALR expression in both HG-3 and OSU-CLL cell lines and primary CLL cells comparable to the known CALR-translocating and ICD-inducing agent etoposide (Figure 1). Importantly, these data substantiate the capacity of CLL cells to proceed with ICD by conferring “eat me” signals to immune cells via ecto-CALR expression.

#### 3.1.2. SpiD3 Treatment Induces ATP and HMGB1 Release from CLL Cells

**Figure 2:**
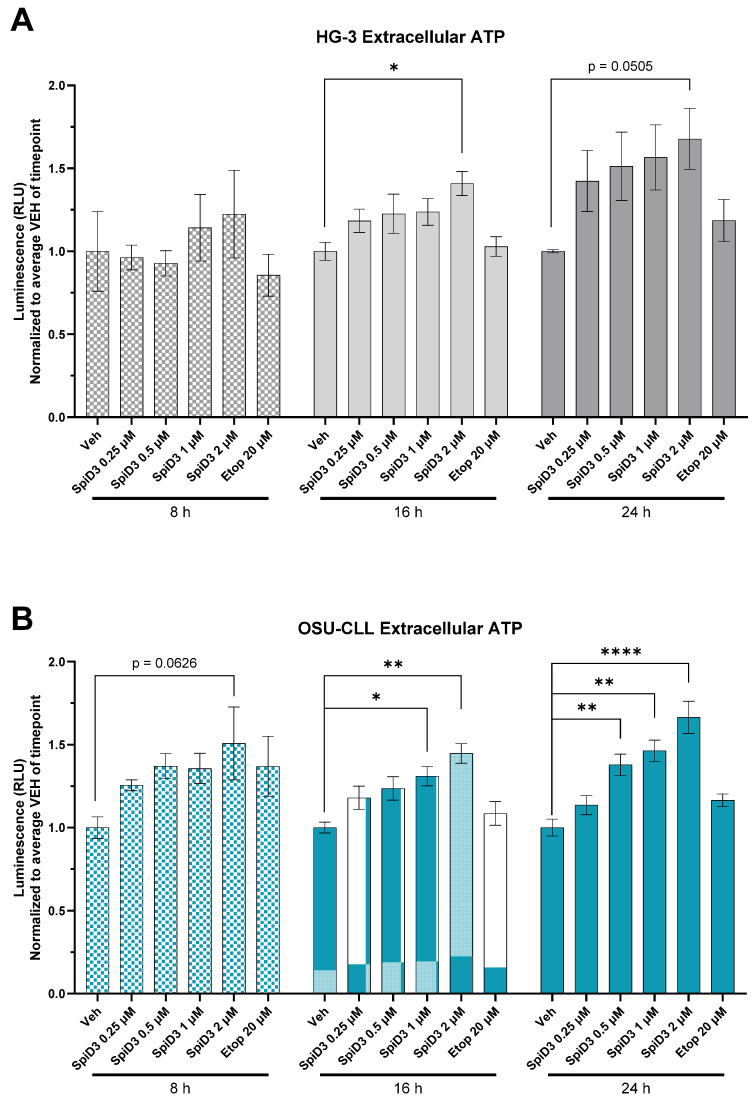
SpiD3 treatment evokes extracellular ATP release. HG-3 (**A**); and OSU-CLL (**B**) cells were treated over 24 h (n = 3) with vehicle (Veh), SpiD3 (0.5–2 µM), or the positive control, etoposide (Etop; 20 µM). Extracellular ATP measurements at 8, 16, and 24 h were parsed out to evaluate the average extracellular ATP measured at these timepoints in comparison to the matched timepoint vehicle. Data are presented as mean ± SEM. Comparisons across treatment groups were analyzed with respect to the matched timepoint average vehicle by one-way ANOVA. Asterisks denote magnitude of significance: * *p* < 0.05, ** *p* < 0.01, **** *p* < 0.0001.

The extracellular release of normally intracellular constituents, such as adenosine triphosphate (ATP) and high-mobility group box-1 protein (HMGB1), has been associated with immunostimulatory contexts and ICD potentiation by facilitating DC recruitment to the site of the dying cell [38,39,40]. The magnitude of extracellular ATP released from HG-3 and OSU-CLL cells due to therapeutic stress was measured over a 24 h treatment period by continuously monitoring ATP-associated bioluminescence (Supplementary Figure S2A,B). SpiD3-treated HG-3 and OSU-CLL cells displayed enhanced luminescence, suggesting a dose-dependent release of ATP into the extracellular space (Supplemental Figure S2A,B). To better investigate how the magnitude of extracellular ATP compared across treatment conditions, the continuous measurements were parsed to obtain average luminescence readings at three timepoints (8 h, 16 h, and 24 h; Figure 2). HG-3 cells treated with 2 µM SpiD3 produced significantly more extracellular ATP than vehicle-treated controls within 16 h (Figure 2A). OSU-CLL cells treated with 2 µM SpiD3 approached statistical significance compared to vehicle-treated controls (*p* = 0.0626) within the 8 h of treatment (Figure 2B). Interestingly, lower concentrations of SpiD3 (0.5 µM and 1 µM), which confer pre-clinical anti-leukemic activity and spare healthy cells [20], also reached statistical significance with a longer duration of treatment (Figure 2B). Extending SpiD3 treatment beyond 24 h did not result in additional extracellular ATP accumulation, for either HG-3 or OSU-CLL cells (Supplementary Figure S2C,D).

**Figure 3:**
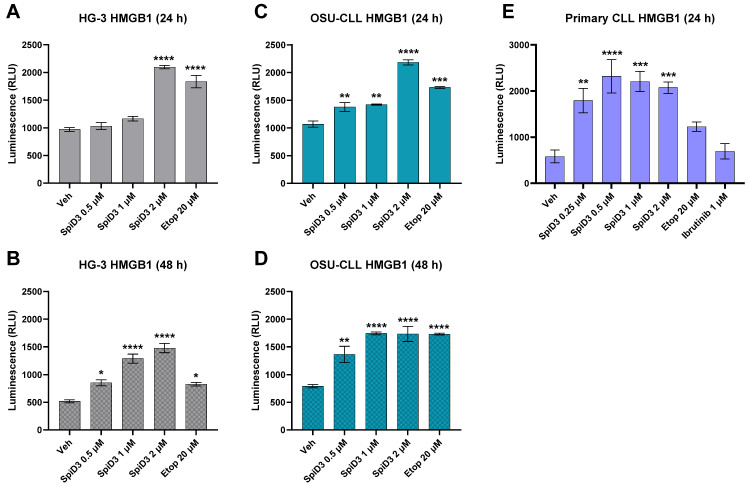
SpiD3-treated cells release extracellular HMGB1. Supernatant from HG-3 ((**A**,**B**); n = 3); OSU-CLL ((**C**,**D**); n = 3); and primary CLL ((**E**); n = 10) cells were evaluated for extracellular HMGB1 after 24 h or 48 h of treatment with the vehicle (Veh), SpiD3 (0.5–2 µM), ibrutinib (1 µM), or positive control, etoposide (Etop; 20 µM). Data are presented as mean ± SEM. Comparisons across treatment groups were analyzed with respect to the vehicle by one-way ANOVA. Asterisks denote magnitude of significance: * *p* < 0.05, ** *p* < 0.01, *** *p* < 0.001, **** *p* < 0.0001.

The magnitude of extracellular HMGB1 released from HG-3 and OSU-CLL cells due to therapeutic stress was monitored after 24 h (Figure 3A,C) and 48 h (Figure 3B,D) treatment periods by measuring HMGB1-associated bioluminescence. HG-3 cells displayed enhanced luminescence with SpiD3 treatment, seemingly dependent on treatment duration (Figure 3A,B). For both CLL cell lines, the magnitude of HMGB1 released into the extracellular space was greatest with 24 h 2 µM SpiD3 treatment, exceeding the magnitude of HMGB1 detected from the positive controls (Figure 3A,C). However, by 48 h of treatment, the magnitude of luminescence detected from 2 µM SpiD3 treatment depreciated, while luminescence values increased following SpiD3 treatment at 0.5 and 1 µM (Figure 3B,D). Interestingly, OSU-CLL cells appeared to release HMGB1 more readily than HG-3 cells, which was demonstrated by the greater magnitude of HMGB1-associated luminescence detected from OSU-CLL cells treated with 0.5 µM or 1 µM SpiD3, compared to their HG-3 cell counterparts (Figure 3).

Although the experimental constraints of kinetic ATP monitoring in this study were not supportive of primary CLL cell viability, SpiD3-induced HMGB1 release from these samples could be evaluated (Figure 3E). Following 24 h of SpiD3 treatment, primary CLL samples demonstrated a statistically significant increase in HMGB1-associated luminescence compared to those of the vehicle and etoposide, while the prominent CLL therapeutic, ibrutinib, was incapable of evoking HMGB1 release (Figure 3E). Together, these data demonstrate that SpiD3 treatment enhanced extracellular ATP and HMGB1 release from CLL cells, in a dose-dependent fashion, substantiating the capacity of CLL cells to establish a chemotactic gradient for immune cell recruitment in the propagation of ICD.

### 3.2. SpiD3 Treatment Cultivates an Immunogenic Milieu

#### 3.2.1. SpiD3 Treatment Establishes a Chemotactic Gradient for BMDCs

Immunogenic DAMPs first serve to establish a chemotactic gradient for DCs, then act as ligands for DC maturation or phagocytosis signals. Hence, we sought to investigate whether these functional properties, required for ICD transduction, could be associated with SpiD3 treatment, given that classical immunogenic DAMPs (CALR, ATP, and HMGB1) were emitted from CLL cells subjected to SpiD3 treatment.

**Figure 4:**
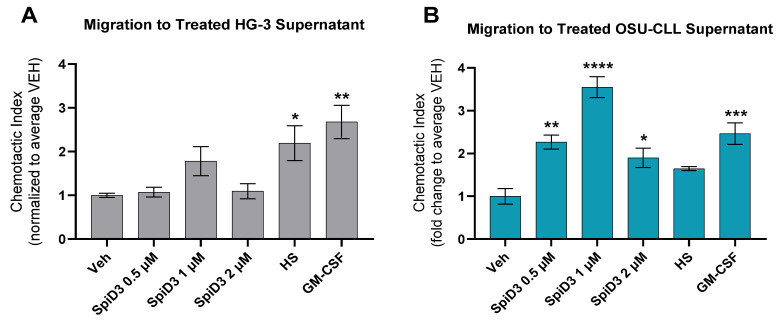
Chemotactic potential of SpiD3-treated cell supernatants. Bone marrow dendritic cells (BMDCs) were allowed to migrate for 6 h toward supernatant collected from HG-3 (**A**); and OSU-CLL (**B**) cells after 24 h treatment with the vehicle (Veh), SpiD3 (0.5–2 µM), or the positive control, etoposide (Etop; 20 µM). GM-CSF (20 ng/mL) stimulated media, and supernatant derived from heat-shocked CLL cells (HS) served as positive chemotactic controls. The number of migrated BMDCs were counted via flow cytometry analysis (n = 3). The chemotactic index is a comparison of the migrated events observed from treatment conditions to that of the vehicle condition. Data are represented as mean ± SEM. Comparisons across treatment groups were analyzed with respect to the vehicle by one-way ANOVA. Asterisks denote magnitude of significance: * *p* < 0.05, ** *p* < 0.01, *** *p* < 0.001, **** *p* < 0.0001.

We developed primary, murine DC populations *ex vivo* by culturing murine bone marrow in GM-CSF- and IL-4-containing media. The generated bone marrow dendritic cells (BMDCs) were allowed to migrate towards supernatant collected from 24 h SpiD3-treated HG-3 (Figure 4A) and OSU-CLL (Figure 4B) cells. The chemotactic index of supernatant from the SpiD3-treated HG-3 conditions did not surpass the chemotactic effect of the positive migration controls (GM-CSF and supernatant from heat-shocked CLL cells; Figure 4A). A greater chemotactic effect was witnessed with supernatants derived from SpiD3-treated OSU-CLL cells (Figure 4). Notably, the chemotactic index of DCs to supernatant from OSU-CLL cells treated at 0.5 or 1 µM SpiD3 demonstrated a magnitude of DC migration and statistical significance rivaling that of the GM-CSF positive control (Figure 4B). SpiD3 (0.5–2 µM) treatment significantly induced extracellular ATP release from OSU-CLL cells as the treatment duration approached 24 h (Figure 3C); the magnitude of HMGB1 released was greatest from OSU-CLL cells treated with 2 µM SpiD3 at this same timepoint (Figure 3C). However, the HG-3 and OSU-CLL cell supernatant conditions with the greatest HMGB1 accumulation (2 µM SpiD3) demonstrated diminished DC migration (Figure 4).

This discrepancy between optimal SpiD3 concentrations for DAMP emission and resultant DC migration may be directly related to the extracellular titer of HMGB1, given that HMGB1 can also suppress the activity of adaptive immune cells in certain contexts [41,42]. Hence, it may be that an overabundance of extracellular HMGB1 is counterproductive to DC migration. Likewise, our secretory DAMP observations in conjunction with the migration data may reflect the importance of balance within the extracellular milieu, given the cytotoxicity associated with 2 µM SpiD3 treatment [20]. Thus, an explanation for diminished DC migration may be that the immuno-potential of the extracellular milieu no longer favors an immunogenic effect due to a significant contribution of non-immunogenic or tolerogenic cell debris in this *in vitro* setting, ultimately masking the presence of immunogenic DAMPs.

#### 3.2.2. SpiD3 Treatment Incites an Immunogenic Response *In Vivo*

**Figure 5:**
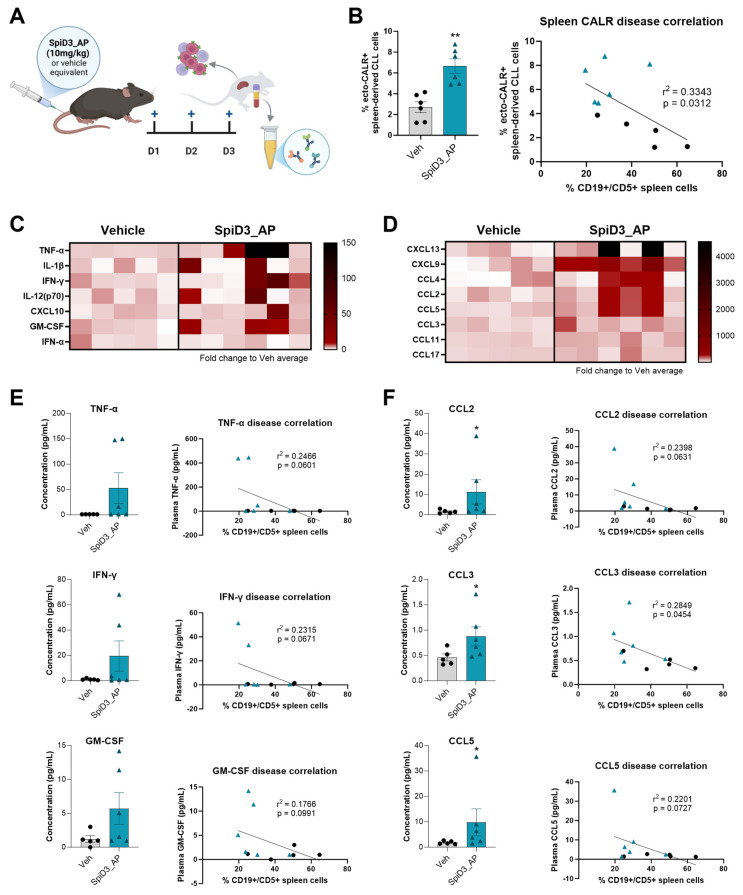
*In vivo* SpiD3 treatment yields an immunostimulatory response. (**A**) Schematic of experiment design: Eµ-TCL1 mice with comparable leukemia burden were treated intravenously with SpiD3 prodrug (SpiD3_AP, 10 mg/kg; n = 6) or equivalent vehicle (Veh; 50% PEG400, 10% DMSO, 40% water; n = 5) once daily for 3 days, as previously reported [20]. Following treatment, spleen cells were collected for flow cytometry analysis and plasma was isolated from murine blood; (**B**) leukemic (CD19+/CD5+) cells from murine spleens were analyzed by flow cytometry for changes in surface CALR expression (ecto-CALR) and compared to the percentage of leukemic cells detected in spleens of the same mice (as reported in Eiken, et al. [20]). The concentrations of plasma inflammatory cytokines and chemokines were assessed using Mouse Anti-Virus Response (**C**,**E**); and Mouse Pro-Inflammatory Chemokine (**D**,**F**) LEGENDplex™ flow cytometry-based multiplex immunoassays. (**C**,**D**) Heatmaps display fold change in the plasma analyte concentration compared to the average of vehicle-treated mice. Columns represent individual mice per treatment group. (**E**,**F**) Raw plasma analyte concentration and correlation with the percentage of CD19+/CD5+ spleen-derived cells are shown for select analytes. Individual data points (Veh = black circles; SpiD3_AP = blue triangles) in addition to summary statistics (mean ± SEM) are shown. Comparisons between treatment groups were analyzed by unpaired *t*-test. Asterisks denote magnitude of significance: * *p* < 0.05, ** *p* < 0.01.

Our previous work demonstrated that mice treated intravenously with a prodrug of SpiD3 (SpiD3_AP, 3 days), which has anti-leukemic effects comparable to that of SpiD3, displayed marked clearance of CLL (CD19+/CD5+) cells in the spleen [20] (Figure 5A). Herein, we sought to assess characteristics of an ICD response following this *in vivo* treatment. Splenic CLL cells from diseased Eµ-TCL1 mice [43,44] treated with SpiD3 displayed a significant increase in ecto-CALR expression compared to their vehicle-treated counterparts (Figure 5B). Moreover, splenic CLL cell ecto-CALR expression negatively correlated with splenic tumor percentage (Figure 5B), suggesting that translocation of CALR to the cell membrane of CLL cells may play a role in their clearance following SpiD3 treatment. Aligning with *in vitro* data, CALR translocation is likely an “early” ICD event, witnessed with only 3 days of SpiD3_AP treatment. Plasma from treated Eµ-TCL1 mice was additionally interrogated for immunostimulatory cytokine and chemokine levels (Figure 5C–F). Plasma from leukemic mice treated with SpiD3_AP, on average, demonstrated increased levels of inflammatory cytokines (Figure 5C) and chemokines (Figure 5D) compared to that of vehicle-treated mice. While not detected at measurable levels in all mice, cytokines indicative of an anti-viral response, such as TNF-α and IFN-γ, were found at higher concentrations in mice treated with SpiD3_AP, and these cytokine concentrations correlated with lower splenic disease burden (Figure 5E). GM-CSF and the chemokines CCL2, CCL3, and CCL5 were additionally detected at higher concentrations in SpiD3_AP-treated mice compared to vehicle-treated mice, which is indicative of monocytic and granulocytic cell recruitment (Figure 5E,F). Thus, *in vivo* treatment may incite an inflammatory response to SpiD3-mediated CLL cell death. Future investigation utilizing immune cell-deficient (e.g., DC, T-cell) murine models and longer SpiD3 treatment durations could help to determine the role of immune cell populations and ICD response on SpiD3 anti-leukemic activity.

## 4. Discussion

**Figure 6:**
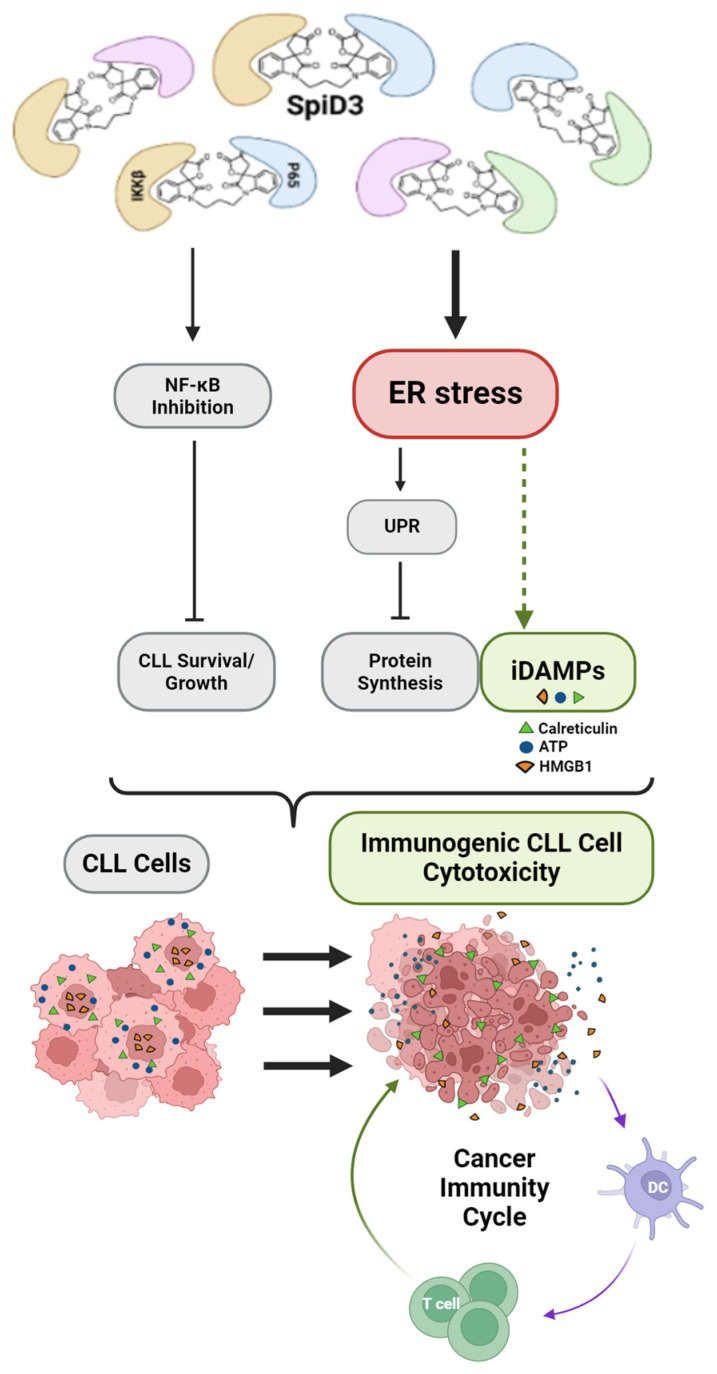
Illustrative summary of SpiD3 anti-leukemic activity. CLL cell cytotoxicity via SpiD3 is demonstrated by: (i) inhibition of NF-κB signaling; and (ii) accumulation of unfolded proteins, promoting ER stress, activating a futile UPR and, subsequently, the associated programmed cell death pathways. ER stress is a proposed prerequisite for immunogenic DAMP emissions; we hypothesize it is this facet of SpiD3-associated effects that result in detectable hallmarks of immunogenic cell death from CLL cells. This diagram is adapted from Eiken, et al. CLL, chronic lymphocytic leukemia; DC, dendritic cell; iDAMP, immunogenic damage-associated molecular pattern; ER, endoplasmic reticulum; UPR, unfolded protein response.

This study demonstrates that treatment with a novel spirocyclic dimer elicited features of an ICD response in CLL cells (Figure 6). SpiD3 treatment encouraged translocation of CALR from the ER lumen to the cell surface membrane in both HG-3 and OSU-CLL cells within 24 h of treatment, in a manner comparable to the known CALR-translocating and ICD-inducing agent, etoposide. Simultaneously, we showed SpiD3 treatment induced extracellular ATP and HMGB1 release from both HG-3 and OSU-CLL cells in a dose- and time-dependent fashion. For both CLL cell lines, the magnitude of extracellular HMGB1 released appeared to be greatest from lower concentrations of SpiD3 (0.25–0.5 µM) in a prolonged treatment setting (beyond 24 h; Figure 3). The chronology of DAMP emission demonstrated by HG-3 and OSU-CLL cells subjected to SpiD3 treatment—starting with ecto-CALR, followed by extracellular ATP and HMGB1 release—is concordant with studies in the literature characterizing ICD in other tumor models [31,45,46]. Our detection of DAMP emission from SpiD3-treated CLL cell lines substantiates the capacity of CLL cells to propagate ICD by: (i) conferring “eat me” signals to engage immune cells; and (ii) establishing a chemotactic gradient for immune cell recruitment through the release of ATP and HMGB1 into the extracellular space. These findings are of particular significance given the low immunogenicity of CLL cells [47,48].

Subsequently, we showed that the chemotactic gradient established by secretory DAMPs released in response to SpiD3 treatment is sustainable for DC migration. This effect was most pronounced when the supernatant was derived from CLL cells treated with lower concentrations of SpiD3 (0.5 µM or 1 µM), which have demonstrated pre-clinical potential for an effective therapeutic window [19,20]. Regardless of the magnitude of ecto-CALR, extracellular ATP, or extracellular HMGB1 detected from 24 h of the 2 µM SpiD3 treatment (Figure 1, Figure 2 and Figure 3), it is likely that these immunogenic DAMPs were masked by concurrently impressive magnitudes of other cell death debris, ultimately diminishing the immunogenic function of DAMPs associated with this potent treatment condition. Additionally, an overabundance of extracellular HMGB1 could be counterproductive to DC migration [41,42], although further experiments would be necessary to understand how HMGB1 titers—in isolation from other cell death debris—influence DC recruitment. Nevertheless, leukemic mice treated with an SpiD3 prodrug demonstrated elevated splenic CLL ecto-CALR expression and plasma immunostimulatory factors suggestive of an immunogenic response to treatment. Additional experiments proving an enhanced DC response to these conditions would be the next step in determining if SpiD3-related DAMP emissions contribute to *bona fide* ICD. Lastly, the gold standard for ICD-inducing agents is a vaccination study to demonstrate that immunologic memory has been queued for tumor-specific antigens and that this immunologic memory would not be otherwise conferred if it were not for the treatment-related DAMP emissions [16,38].

Previously, we have shown that SpiD3 targeting of surface-exposed cysteine residues promotes futile activation of the UPR in CLL cells through immense misfolded-protein mimicry, culminating in insurmountable ER stress and ferroptotic CLL cell death [19,20]. Notably, cancer cells, including CLL cells, experience higher oxidative stress from ROS than non-malignant cells, which can be compensated for through the maintenance of the antioxidant glutathione [49,50,51,52]. Under the oxidative stress conditions imposed by elevated ROS, glutathione synthesis can be sustained by the increased uptake of L-cysteine, predominantly in its disulfide form, L-cystine [49]. As such, depletion of the extracellular L-cysteine and L-cystine pools using a human cyst(e)inase enzyme selectively prompted prostate cancer cell cycle arrest and death by subsequent depletion of intracellular glutathione and ensuing elevated ROS [49]. Elevated glutathione levels and reliance on this antioxidant has also been shown to enhance CLL survival, contributing to protection from drug-induced cytotoxicity [52]. Novel strategies that disrupt glutathione synthesis in CLL may generate unreconcilable ROS, leading to immunogenic DAMP emissions along with CLL cell death. It is possible that SpiD3 contributes to immunogenic CLL cell death in this way, as spirocyclic dimers have been shown to: (i) generate ROS production from CLL cells; and (ii) covalently modify surface-exposed cysteine residues through the Michael acceptor moiety, leading to misfolded protein mimicry and futile UPR activation [20,22,23]. Given its covalent interactions with surface-exposed cysteine residues [22,23], SpiD3 treatment may also interact with L-cysteine and L-cystine, during which the covalent interactions would sequester these molecules from glutathione synthesis mechanisms, resulting in intracellular ROS accumulation and susceptibility to oxidative stress as the glutathione levels diminish. Further investigation is needed to determine if SpiD3 treatment directly affects L-cysteine, L-cystine, and glutathione to generate ROS and immunogenic DAMP emissions in CLL cells.

Interestingly, SpiD3 treatment may confer additional means of adaptive immune cell engagement beyond ICD. Covalent drugs binding to mutated residues have been shown to generate hapten-peptides (neoantigens) that are presented by MHC class I for the subsequent activation of cytotoxic T-cells [53,54]. Future studies could determine if SpiD3 treatment will not only induce immunogenic DAMP emissions for ICD, as described here, but also result in the generation of unique MHC-I-restricted neoantigens that can engage cytotoxic T-cells for better tumor control, which is again of particular significance given the low mutational burden and poor antigenicity of CLL cells [47,48].

## 5. Conclusions

In the case of CLL, durable clinical responses with (immuno)therapy have been stifled by: (i) innate and adaptive immune suppressive mechanisms that promote immune tolerance and CLL immune evasion; and (ii) the tumor’s overall poor antigenicity/low immunogenicity, which limits the development of anti-tumor immunity. We have shown that CLL cells (*in vitro* and *in vivo*) have the capacity to propagate ICD through emissions of classically immunogenic DAMPs under SpiD3 treatment, and that the secretory DAMPs establish a viable chemotactic gradient for BMDCs. However, immunogenic DAMPs can be confounded by immunosuppressive DAMPs (e.g., adenosine, PGE2) [55,56,57,58]. The pro-tumor microenvironment of CLL is already sustained by suppressive soluble factors (e.g., IL-10, TGF-β) and inhibitory immune molecules (e.g., PD-L1, LAG3), among which the tolerogenic milieu [59,60,61,62,63,64] would only be compounded by therapeutics that induce immunosuppressive DAMP release from CLL cells. Thus, novel therapeutics that specifically induce immunogenic DAMPs, or that can be titrated to achieve a net-immunogenic DAMP emission profile, might shift the microenvironment towards one that is more supportive of adaptive immune cell engagement.

Due to the innately tolerogenic milieu of CLL, it may also be necessary to consider other interventions—besides immunogenic DAMP-inducing agents— to ultimately propagate ICD and sustain the cancer-immunity cycle. For example, although CLL cells may be perturbed to emit immunogenic DAMPs by therapeutic agents, the surrounding tolerogenic milieu may mask immunogenic DAMP emissions from engaging immune cells. Similarly, even if immunogenic DAMPs are sufficiently sensed by DCs, it may be that T-cell-directed killing is still not achievable due to CLL-acquired T-cell dysfunction [60,61,62,65]. Therefore, in addition to promoting immunogenic DAMP emissions, the neutralization of tolerogenic DAMPs or attempts to revitalize T-cells may also be important approaches to propagate ICD and sustain the cancer-immunity cycle.

## 6. Patents

A.N. and S.R. hold a patent for SpiD3 as a novel dimer of covalent NF-κB inhibitors (US 2019/0322680 A1).

## Data Availability

Data will be made available from the authors upon reasonable request.

## Supplementary Materials

The following supporting information can be downloaded at: https://www.mdpi.com/article/10.3390/biomedicines12122857/s1, Methods Figure S1: Representative flow cytometry plots illustrating gating strategy for detection of peroxidized lipids; Methods Figure S2: Flow cytometry gating strategy for CALR detection on CLL cell lines and primary patient-derived or Eμ-TCL1 CLL cells; Table S1: Characteristics of CLL patient-derived samples used throughout the study; Figure S1: Peroxidized lipid generation following SpiD3 treatment; Figure S2: Extended extracellular ATP monitoring.
