# Supplementary material for: Immunogenic Cell Death Traits Emitted from Chronic Lymphocytic Leukemia Cells Following Treatment with a Novel Anti-Cancer Agent, SpiD3
Source: Biomedicines. 2024 Dec 16;12(12):2857. doi: 10.3390/biomedicines12122857 (PMC11673838; doi:10.3390/biomedicines12122857)
Supplement: Supplementary file 1 [file biomedicines-12-02857-s001.zip › biomedicines-3366343-supplementary.pdf]

Supplementary File

I. Supplementary Methods

Ferroptosis detection: HG-3 and OSU-CLL cell lines were seeded at 1e6 cells/mL in a 24-well plate and allowed to incubate for 1 hour with or without 10 μM of the ferroptosis sparing agent, ferrostatin-1. Following pre-treatment, cells were treated with vehicle (DMSO), SpiD3 (0.5 - 2 μM), etoposide (20 μM), or 160 μM iron (II) chloride tetrahydrate ($FeCl_2$) for up to 48 hours at 37°C. Ferroptotic cell death was monitored by flow cytometry using Zombie NIR (Cat #423106; BioLegend) for live/dead discrimination in combination with lipid peroxidation discrimination using the BODIPY-C11 (BC11) 581/591 probe (Cat #D3861; Invitrogen). Lipid populations (reduced: PE channel; oxidized: FITC channel) were identified in comparison to cells stained with Zombie NIR viability dye alone. The gating strategy is included below in **Supplementary Methods Figure 1**. Changes in lipid peroxidation from viable cells were monitored by the percentage of PE+/FITC+ events observed in comparison to the matched vehicle-treated condition.

Extracellular ATP measurement: For 48-hour treatment assays, HG-3 and OSU-CLL cell lines subjected to treatment with vehicle (DMSO), SpiD3 (0.5 - 2 μM), or etoposide (20 μM) were incubated in RMPI-1640 media for an initial 24 hours at 37°C. Cells were then transferred to CO_2 independent media (Leibovitz's L-15 Medium; Cat #11415064; Gibco), re-seeded, and re-treated in a luminescence-grade 96-well plate (Cat #655095; Greiner-Bio) as described in main text **Methods** for subsequent 24-hour kinetic monitoring of extracellular ATP.

Flow Cytometry Gating Strategies

Supplementary Methods Figure 1. Representative flow cytometry plots illustrating gating strategy for detection of peroxidized lipids.

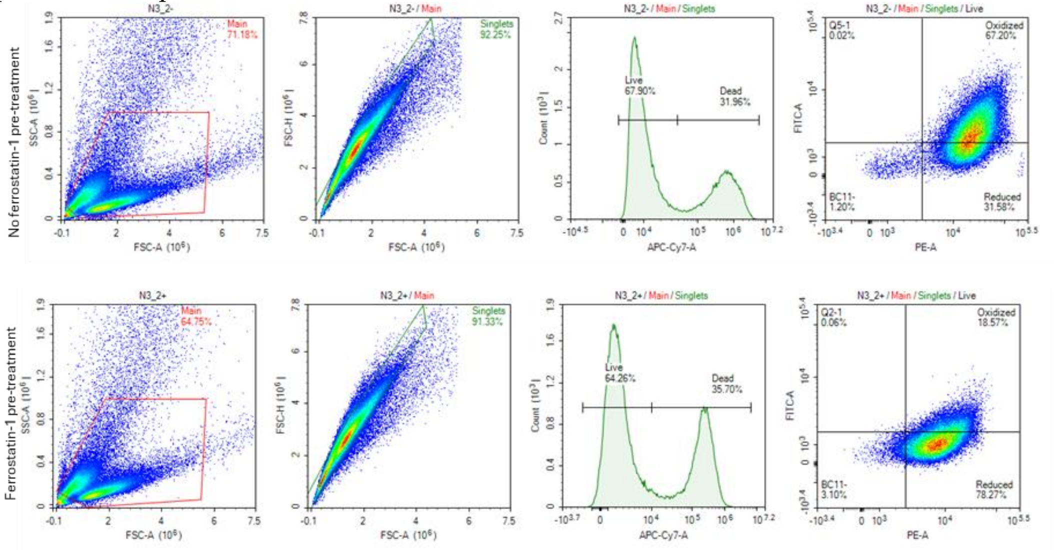

Supplementary Methods Figure 2. Flow cytometry gating strategy for CALR detection on CLL cell lines (top) and primary patient-derived or Eμ-TCL1 CLL cells (bottom).

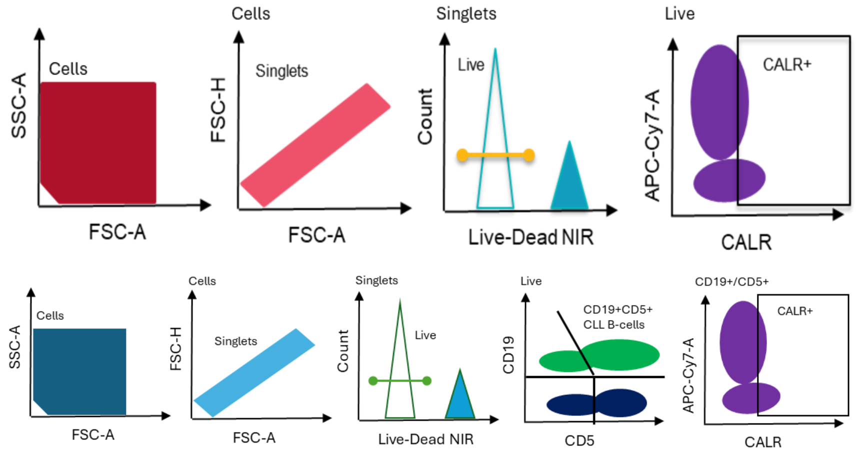

II. Supplementary Tables

ID #	Gender	Age	IGHV mutational status	Treatment status	FISH Cytogenetics					Karyotype	Figures
					del 13q	del 17p	del 11q	del 6q	tris 12	Normal or Complex (≥3 abnormalities)	
pt.1	M	59	Unmutated	Naïve	+	-	+	-	-	NA	1E-F, 2E
pt.2	M	70	NA	Naïve	+	-	-	-	-	Normal	1E-F, 2E
pt.3	M	48	Unmutated	TT	+	-	-	-	-	Complex	1E-F, 2E
pt.4	M	78	Mutated	Naïve	+	-	-	-	-	Normal	1E-F, 2E
pt.5	F	57	Unmutated	Naïve	+	-	+	-	-	NA	1E-F, 2E
pt.6	F	82	NA	TT	+	-	-	-	-	Normal	2E
pt.7	F	68	Mutated	TT	+	-	-	-	-	Normal	2E
pt.8	M	79	Mutated	Naïve	+	-	-	-	-	Normal	2E
pt.9	M	68	Unmutated	Naïve	-	-	-	+	-	NA	2E
pt.10	M	67	Unmutated	Naïve	+	+	-	-	-	Normal	2E

Supplementary Table 1. Characteristics of CLL patient-derived samples used throughout the study. IgHV, immunoglobulin heavy chain variable region; TT, treated; FISH, fluorescence in situ hybridization; del, deletion; tris, trisomy; M, male; F, female; NA, not available.

III. Supplementary Figures

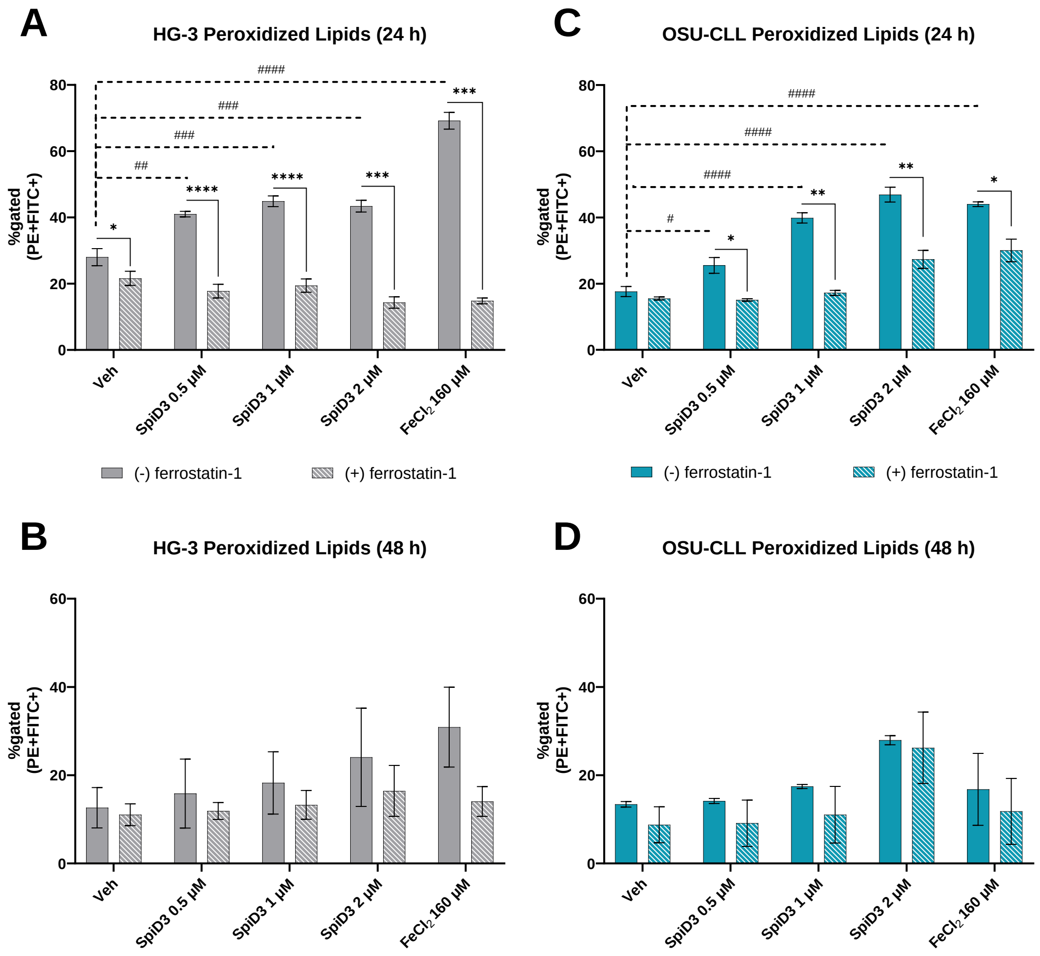

Supplementary Figure 1. Peroxidized lipid generation following SpiD3 treatment. HG-3 (**A-B**), and OSU-CLL (**C-D**) cells were treated for 24 hours (n = 3) or 48 hours (n = 2) with vehicle (Veh), SpiD3 (0.5 - 2 µM), or 160 µM $FeCl_2$ with or without 1-hour pre-treatment with 10 µM ferrostatin-1. Cells were analyzed by flow cytometry for changes in peroxidized lipid generation, observed by a change in fluorescence from PE to FITC. $FeCl_2$ serves as a positive, lipid peroxidation control. Data are presented as mean ± SEM. Within-group (solid lines) comparisons were analyzed by unpaired t-test. Comparisons across treatment groups (dotted lines) were analyzed with respect to the matched vehicle by one-way ANOVA. Asterisks and hash symbols denote magnitude of significance: */# $P < 0.05$, **/## $P < 0.01$, ***/### $P < 0.001$, ****/#### $P < 0.0001$.

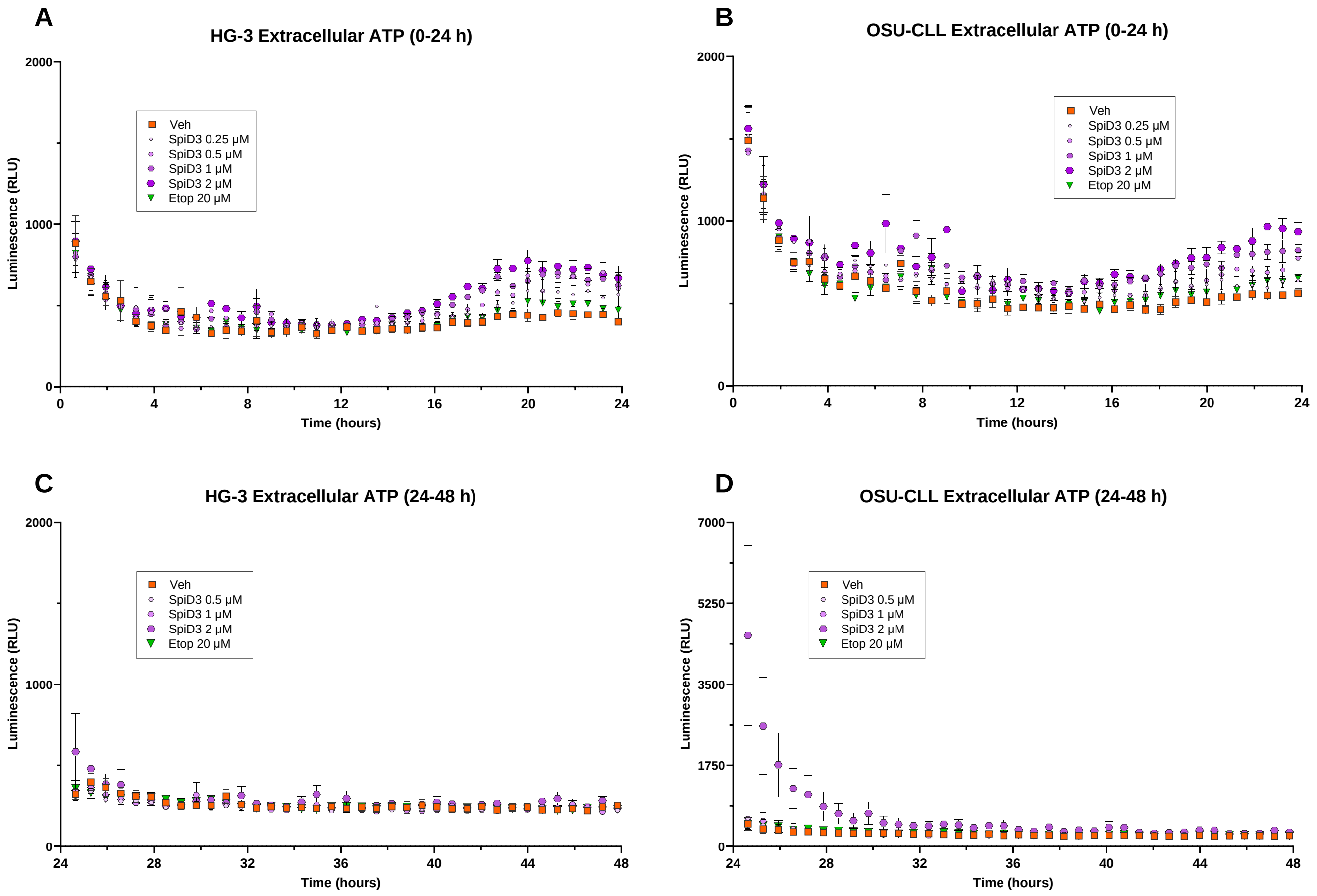

Supplementary Figure 2. Extended extracellular ATP monitoring. HG-3 (**A-B**) and OSU-CLL (**C-D**) cells were treated over 24 or 48 hours (n = 3) with vehicle (Veh), SpiD3 (0.5 - 2 µM), or positive control, etoposide (Etop; 20 µM). ATP-associated luminescence was continuously measured over the total or final 24-hour time course. Data are presented as mean ± SEM.
